# Toward Standardized Guidelines for Investigating Neural Circuit Control of Behavior in Animal Research

**DOI:** 10.1523/ENEURO.0498-20.2021

**Published:** 2021-04-19

**Authors:** Alan S. Lewis, Erin S. Calipari, Cody A. Siciliano

**Affiliations:** 1 Department of Psychiatry and Behavioral Sciences, Vanderbilt University Medical Center, Nashville, TN 37212; 2 Vanderbilt Brain Institute, Vanderbilt University, Nashville, TN 37232; 3 Department of Pharmacology, Vanderbilt University, Nashville, TN 37232; 4 Vanderbilt Center for Addiction Research, Vanderbilt University, Nashville, TN 37232

**Keywords:** behavior, behavioral neuroscience, chemogenetics, circuits, *in vivo* imaging, optogenetics

## Abstract

With the advent of tools for recording and manipulating activity with high spatiotemporal resolution in defined neural circuits in behaving animals, behavioral neuroscience is now tasked with establishing field-wide standards for implementing and interpreting these powerful approaches. Theoretical frameworks for what constitute proof of fundamental neurobiological principles is an ongoing and frequently debated topic. On the other hand, standardizing interpretation of individual experimental findings to avoid spurious conclusions in practice has received less attention. Even within subfields, similar assays are often used to support widely disparate conclusions which in part has contributed to a slew of studies claiming highly specified functions for cell types and circuits which are often in direct disagreement with one another. In this opinion piece, we discuss common pitfalls in design and interpretation of approaches for recording or manipulating neural activity in animal models of motivated behavior. We emphasize the importance of integrating findings across multiple behavioral assays concomitant with tempered inference regarding specialized neuronal functions as a standardized starting point for parsing circuit control of behavior. Our aim is to stimulate an open and accessible discourse in the literature to address issues of continuity across behavioral neurosciences.

## Significance Statement

New technologies in neuroscience allow for increasingly precise recording and stimulation of neural circuits in animal research. The goal is to understand the function of the brain, as well as to develop new ways to treat brain diseases. Yet despite seemingly boundless technical potential, a key limitation for meaningful advances stems from spurious interpretation of experiments. Here, we provide specific examples from our own studies and discuss why appropriate interpretation of results can be challenging. We emphasize that standardizing interpretation of behavioral assays, as well as transferring incentives for identifying “specific” circuits to more objectively understanding a circuit’s role across varied behavioral domains, will facilitate assimilation across literatures and ultimately move behavioral neuroscience closer to its goals.

## Introduction

Neuroscientists have never before enjoyed a more powerful repertoire of techniques to manipulate the *in vivo* activity of cells and circuits in the experimental animal brain. These methods, which seem to advance in complexity and utility almost daily, enable precise observation or control of activity in identified cells using developmental, genetic, functional, and/or connectivity classifications. These techniques have overwhelmingly been used to relate the activity of a particular region, circuit, or cell-type to animal behavior. With these data, broad conclusions are often made claiming a highly specified role for circuits controlling certain aspects of behavior or encoding specific features of experience. While this is tempting and, on its surface, logical, a multitude of factors contribute to observed behavior, which means that manipulating any number of latent variables may alter a behavioral output in the same way. In this opinion piece, our goal is to reconcile the increasingly widespread use of *in vivo* manipulation techniques with the need for the field of behavioral neuroscience to establish greater standardization for labeling a manipulation as truly “specific.”

## A Cautionary Anecdote

One of the authors recently conducted a series of studies to test the effects of mossy cells in the mouse ventral dentate gyrus on social isolation-induced aggression using the resident-intruder test, a commonly used assay for territorial aggression (Koolhaas et al., 2013). Ventral mossy cells were activated by expression of the designer receptor exclusively activated by designer drugs (DREADD) hM3DGq and systemic administration of 10 mg/kg of the DREADD ligand clozapine N-oxide (CNO; Armbruster et al., 2007; Roth, 2016). When compared with control mice expressing only the fluorophore mCherry, reduction in aggression was observed in hM3DGq-expressing mice, suggesting that ventral mossy cell activation causally inhibited aggression. However, examination of protein expression of the immediate early gene cFos following CNO administration showed not only the expected dense cFos expression in hM3DGq-expressing mossy cells, but also in non-hM3DGq-expressing granule cells and pyramidal neurons of CA3 and CA1 throughout the hippocampal formation, not seen in control conditions. This pattern of dense cFos expression is commonly observed in rodents following seizure induction (Chawla et al., 2005; Peng and Houser, 2005), and even subtle behavioral seizure induction from overactivation of the ventral dentate gyrus might explain reduced aggression. Indeed, careful review of videotaped behavioral assays confirmed mild behavioral seizures in a subset of mice during resident-intruder interactions. Thus, while our initial conclusion that ventral mossy cell activation was sufficient to reduce aggression in mice was logical based on our behavioral findings, our subsequent cFos and behavioral analysis suggested that (at least using 10 mg/kg CNO) an intervening behavior, in this case myoclonus, likely was causal for aggression reduction.

This anecdote highlights a particularly important caveat of neural manipulation studies: a manipulation reducing or increasing a behavior in a single task is not sufficient to claim that circuit encodes a specific experience or is specific in controlling a behavior. For any of the individual tasks that we use in behavioral neuroscience there are many variables that can change task performance. Thus, it is critical that we describe data based on the effects that manipulations have on a given behavioral measure, rather than as generalizations focused on the information encoded within circuits without ruling out hidden variables and alternative explanations.

## When Does Causality Matter?

**Figure 1.**
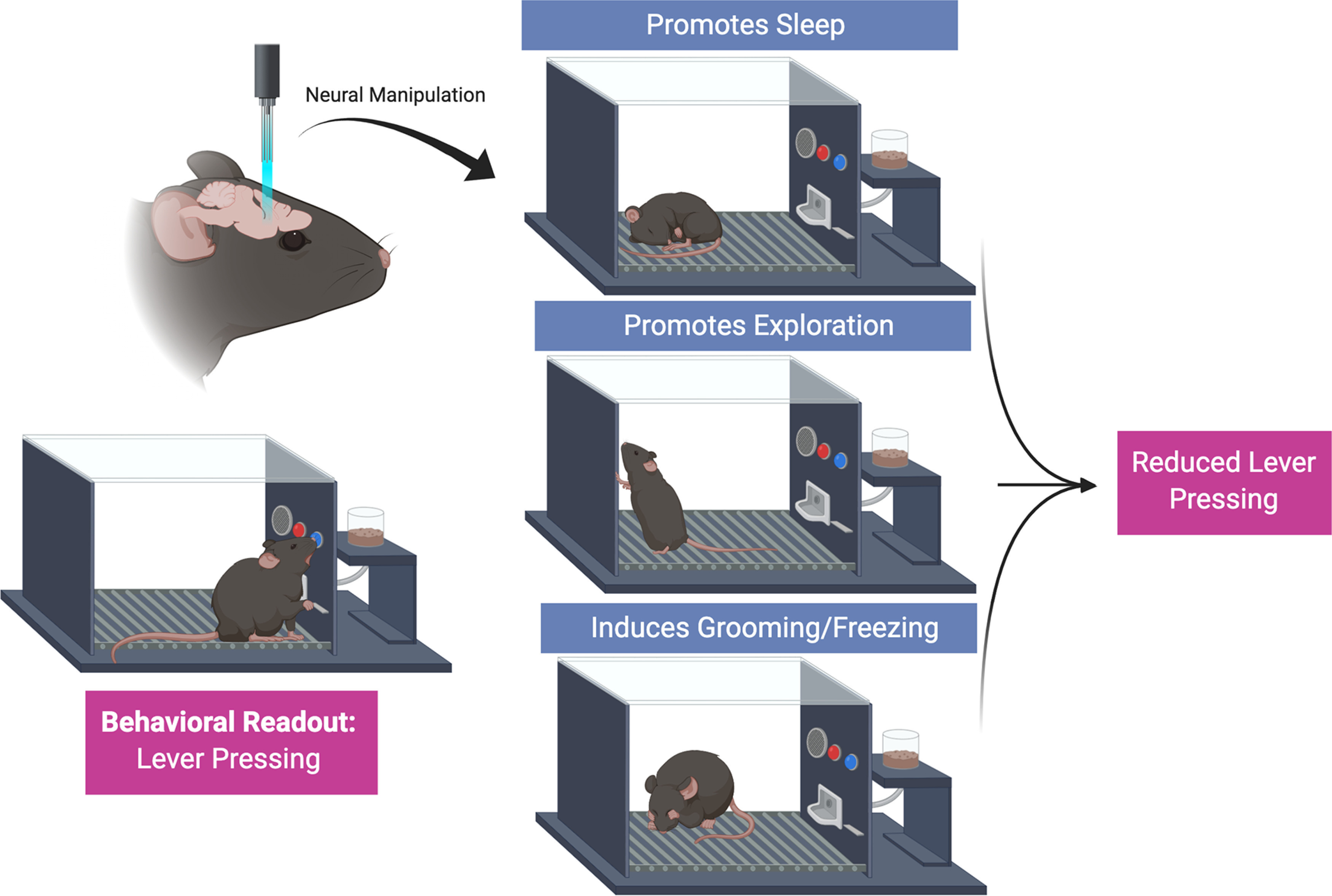
The pitfalls of making conclusions implicating latent variables from individual behavioral measures. Behavior is driven by a range of competing factors that act in unison to drive specific behavioral outputs at specific times. In this example, the dependent measure is lever pressing. In each case, it would be accurate to say that the manipulation of the neural circuit reduced lever pressing. However, this occurs for different reasons in each case. Thus, making the conclusion that the neural circuit encodes appetitive responding or motivation would be inaccurate. For experiments like these, it is important for the field that we have continuity in design and interpretation (i.e., “lever pressing is reduced”) than the semantics of what a circuit encodes. This will allow for accurate data to accumulate across behavioral tasks and conditions to facilitate precise conclusions about how circuit manipulation controls behavior across contexts.

The need to go beyond describing the effect of circuit manipulations as a function of how they alter a behavioral output depends on the ultimate goals of the researcher. Translational researchers whose motivation is to target and regulate behaviors of clinical importance might focus more on demonstrating “whether” a circuit manipulation influences a behavior and less on “why” and “how” it works. For instance, consider a hypothetical neural manipulation that reduces lever pressing for cocaine in rodents. The manipulation may influence attention, salience, pharmacodynamic, or motivational processes among many others, any one of which could reduce cocaine taking in preclinical behavioral assays (Fig. 1). If the equivalent neural manipulation in humans (for example, via pharmacological or brain stimulation methods) also reduces cocaine use in patients with cocaine use disorder, then the important question is not whether the manipulation is highly specific for cocaine taking, but rather whether any negative consequence of the manipulation outweigh the clinical benefit.

For experiments with the goal of using contemporary tools to precisely define the information encoded within a defined circuit, it is critical to consider how many variables might influence a given behavior. In the example above, there was no question that the manipulation reduced aggression, but it did so presumably because the behavior was interrupted by mild motor seizures rather than an isolated effect on aggression. During any behavioral task, salience, novelty, value, predictions, action initiation/motor responses, sensory information, and many other processes are simultaneously contributing to temporally-specific neuronal activity signatures around discrete behavioral events. Manipulating circuit activity to change how information is processed could alter behavior because of effects on any one or many of these factors. In fact, learning-theory models that provide a formalized framework for how organisms use information to make decisions show that the computations are flexible such that any given change in learning rate/performance could be achieved through many different distinct combinations of alterations in underlying variables (Rescorla et al., 1972; Sutton and Barto, 1987). Thus, assuming an experimental manipulation altered a given latent variable (perceived stimulus value or valence, for example) based on an observed change in learning rate is tenuous given that manipulation of other variables (e.g., perceived stimulus salience) could result in an identical change in performance. If one is testing the hypothesis that a circuit encodes the valence of a reward-paired cue and Pavlovian conditioned approach behavior is reduced when the circuit is inhibited, one might conclude that the circuit does encode valence and that reduction in approach reflects decreased attractiveness of the cue. However, considering this effect in isolation, it is equally likely that the circuit encodes salience given that inhibition would also reduce approach behavior, even if valence is unaffected, simply because the animal is less likely to notice the cue presentation.

Many of the behavioral tasks used in circuit manipulation studies were designed to answer questions about pharmacology, translational value, or general behavior, not to parse the underlying co-occurring variables that could contribute to the effects of a circuit manipulation on behavior. It is therefore paramount to consider how to disentangle these many factors to develop durable, reproducible conclusions. Complex tasks have been designed and continue to be developed for this purpose, but accessible dissemination of these approaches to facilitate implementation by researchers with diverse expertise and interests will require sustained effort. Batteries of relatively simple behavioral tasks can also be used in combination to count out common pitfalls. Together, the field must think carefully about how best to incorporate these considerations into research funding, experimental execution, and publication to move forward.

## Why We Need Standardization

Insisting on purely descriptive reporting of behavioral findings, without inference of underlying mechanisms or implications for human behavior, can easily be perceived as pedantic and overly conservative. While descriptive reporting of behavioral results taken to an extreme can limit the utility of using animals as models of human behavior and for studying neurobiology in general, a lack of standardized frameworks for interpreting results from behavioral assays is not without consequence. Take fear conditioning, for example, a commonly used task that has relatively standardized methodology across laboratories. Despite similar methodology for performing the assay, there are widely disparate conclusions made from nearly identical experimental findings across subfields. Many studies have shown that various manipulations of hippocampal activity result in alterations in freezing behavior when animals are presented with a shock conditioned cue; typically, this result is attributed to changes in memory recall. When manipulations are made in the amygdala, the same experimental result of increased or decreased freezing behavior is often attributed to changes in fear responses. In other regions, the same experimental result has been used to make conclusions regarding circuit control of valence coding or threat responses. It is possible that none of these conclusions are incorrect, it is reasonable to think that changes in memory recall could affect a fear response, or that threat, fear, and valence coding are inextricably intertwined. Nevertheless, the fact that the exact same behavioral readout can be attributed to disparate latent variables depending on the subfield or hypotheses of the experimenter is problematic.

This issue is not specific to the example above, and similar scenarios can be seen throughout neuroscience research including previous publications from the authors. To give a few more examples, reductions in sucrose preference are often attributed to anhedonia in studies focused on depression, while the same effect may be attributed to appetite in the feeding field, or taste in gustatory studies. Along the same lines, conditioned place preference is used to measure drug reward in the addiction field and reconsolidation in the memory field, while marble burying is used to measure anxiety, compulsive behaviors, and autism-like behaviors across studies. Although many studies have run the appropriate controls and no particular conclusion is necessarily at fault, the behavioral neuroscience literature is becoming increasingly difficult to navigate and reconcile.

## For Now, Incremental Steps May Be the Most Impactful

The near infinite combination of underlying processes that influence behavior juxtaposed with the relatively few behavioral outputs that are quantified in a typical experiment creates logical snares which are conceptually attractive and difficult to avoid. Indeed, the ratio of latent variables to measured behaviors produces a scenario whereby any conclusion attributing an underlying cause that cannot be directly measured (e.g., the intent to commit aggression) to a circuit based on the outcome of a single experiment is almost certain to be a “fallacy of the converse.” Fallacy of the converse, or affirming the consequent, describes the error of using a valid if-then statement (if this circuit controls aggression → manipulating its activity will alter aggressive behavior) to support its converse (manipulating the activity of this circuit altered aggressive behavior → this circuit controls aggression). The multiplicity of brain-behavior relationships combined with the robustness with which modern neuroscience tools alter behavior make this fallacy all too easy to commit, and the task of interpreting basic experiments quickly becomes daunting when considering the complexity of these relationships.

While grappling with defining ground-truths of cause-and-effect in neuroscience is no doubt a critical endeavor, we propose that for most behavioral neuroscientists, whose interests lie in specialized subfields, it is important to be aware of these issues but that the immediate goal should be practical, easily applicable mitigation strategies. There seems to be a growing awareness of how often our claims of neural circuit function are incorrect or incomplete, even when supported by evidence that would be considered iron-clad just a few years ago; it is important to acknowledge that it is not practical to wait for paradigm-shifting technical approaches or conceptual frameworks to correct these issues en masse. In this spirit, although none of the authors have expertise in theoretical neuroscience, or claim to have any ground-breaking concepts to present, we wrote this piece aiming to articulate a few common pitfalls that we have learned the hard way as simple guidelines that can easily be understood and applied across subfields. If relatively minor but widely applicable guidelines are continued to be put forward by researchers with diverse expertise in an open forum it could have a major impact on the field as a whole.

## What To Do in Practice

While we are far from the first to bring up interpretational pitfalls introduced by the application of powerful new technologies, much of the literature in this area has focused on creating frameworks for fundamental brain functions, how neural activity allows for complex computation, and constructs to define what is and is not causality. Consensus on these issues is critical for moving neuroscience forward, but debate on this level can quickly become abstract and even philosophical in nature. Practical issues of design and interpretation are more often debated in conference poster sessions and in peer-review. This is in part because of the fact that a universally applicable framework for interpretation of experimental results is almost certainly unachievable and therefore difficult to begin to address in published works: each new question and experimental finding may lead to entirely new conceptual areas or follow-up experiments, and exact methodology will always vary across laboratories. However, we posit that the impossibility of universal rules does not preclude the utility of openly discussing general guidelines for standardizing how conclusions are drawn from commonly used techniques and experimental designs.

We aim to facilitate this discussion by briefly proposing a few practical steps that can easily be incorporated in any behavioral neuroscience lab and in dissemination of findings.

## Eyes on the Behavior

Quantitative measures are of utmost importance for reporting data, but there is no replacement for the human eye for drawing valid conclusions from behavioral data. Indeed, many of the pitfalls described above can be easily avoided by simply observing the animals’ behavior during the experiment. To give an example, one of the authors conducted a conditioning experiment where an auditory cue was associated with the delivery of electrical footshock. Instead of learning that the cue predicted an unavoidable aversive stimulus, some subjects learned to cling to the wall of the conditioning chamber to avoid the electrified floor, and instead used the cue to time their jump and return to the floor once the absence of the cue signaled safety. Because animals were immobile while hanging from the wall, an automated analysis would likely lead the experimenter to conclude that the subject was freezing in anticipation of the shock and had acquired a conditioned fear response as was intended. Observing a neural signal to the cue in this case would lead to potentially spurious conclusions of fear encoding, although it is just as likely that neural activity would be related to escape/avoidance or climbing.

In addition to avoiding the spurious conclusions described here (Fig. 1), observation can also often lead to new discoveries that may not be captured by automated quantification. Importantly, we are not proposing that every experiment requires expensive cameras and time-consuming analytical approaches to quantify continuous behavioral data; rather simple observation can be achieved with inexpensive webcams which can often be interfaced with existing data acquisition setups. In many cases, these data may not even require reporting in manuscripts if no issues are found.

## Results of Any Single Behavioral Effect Should Be Reported Descriptively and Separate from Speculation Regarding Brain-Behavior Relationships

Even the most carefully designed and executed experiments can result in incorrect conclusions, and no number of guidelines and standards will avoid incorrect conclusions from making their way into the literature. However, as authors and reviewers, we can take steps to reduce the impact of potentially spurious or overgeneralized conclusions on the direction of the field. We posit that an underlying and easily correctable issue is using single behavioral effects as evidence to draw conclusions about functional properties of circuits.

For example, instead of reporting that “a real-time place preference task demonstrated that the circuit of interest conveys a positive valence signal” it is more appropriate to first report that “animals spent more time in the circuit manipulation paired chamber in a real-time place preference assay.” That is not to say that speculation is not important. Discussing what an experiment may mean in the context of the greater literature is an important aspect of moving science forward. Drawing more overarching conclusions about the meaning of these data certainly has its place, however, we recommend that these statements should be made separately such that lines between data reporting and speculation are not clouded.

## Reduce Emphasis on Specificity, and When Claims of Specified Circuit Functions Are Made, They Should Be Supported by Systematic Testing across Diverse Behavioral Procedures

“The data are the data.” Coming to terms with this fact and embracing it is a rite of passage for all scientists. However, the attraction of concluding highly specialized functions in the cellular control of behavior is understandable and impact of this drive can be seen throughout the literature, the authors’ work included. This tendency is reinforced by publishing and funding structures where bold claims of particular circuits controlling specific aspects of behavior are often considered to be the most impactful findings. The concern with this approach is that while such discoveries could certainly drive brain understanding forward, the emphasis on the necessity of specificity for widely read journal publication may in fact achieve the opposite by luring investigators to turn a blind eye toward plausible yet less specific explanations for a given behavior. Establishing unified and standardized behavioral controls may circumvent this concern, enabling the reader and reviewer to gain a more holistic understanding of how underlying behavioral processes might hierarchically influence other behaviors. To be clear, we are not prescribing that neuroscientists studying behavior “X” must always perform control experiments “Y” and “Z.” There is simply too much heterogeneity across subfields for this to be practical, and such an approach might even be regressive. Rather, we suggest that the conclusions from specific behavioral tasks should be increasingly standardized with inherent acknowledgment that the lack of evidence for a behavioral confound is not evidence for specificity.

Importantly, when making claims that a circuit’s function is specific to a certain behavior, or controls a behavioral category (e.g., the circuit encodes aggression) that is broader than the dependent variables of the study (e.g., activation of the circuit decreased aggressive behavior in the resident-intruder test), the onus should be on the authors to systematically identify and rule out alternative possibilities. First, clear evidence should be presented as to what underlying processes could potentially alter the dependent behavioral measures used (in most cases these factors have been exhaustively described in the animal behavior and psychology literatures). Once a clear framework for the behavioral category has been established, experimental evidence occluding the contribution of the circuit in question to processes which could also alter the primary dependent behavioral variables should be provided.

## Conclusions

Neural circuit techniques have vaulted neuroscience forward by expanding our ability to manipulate precise cells and circuits in the brain in awake and behaving animals. However, with every technique comes caveats to consider. Here, we have given specific examples from our own groups that highlight how the complex factors that combine to influence behavior can lead to inaccurate assumptions about what neural circuit manipulations mean for behavioral execution. We have outlined some simple practices that can help make conclusions more reproducible: watching behavioral assays, reporting data in precise terms that are separate from speculation, reducing the emphasis on defining specificity, and manipulating circuits across a wide range of procedures. Together, these approaches will rule out alternative latent variables and facilitate generalizability of conclusions across fields. As we move forward, we need to have these debates formally in the literature to gain consensus across fields and better support these scientific goals as a community.

## Synthesis

Reviewing Editor: Michael Michaelides, NIDA-NIH Decisions are customarily a result of the Reviewing Editor and the peer reviewers coming together and discussing their recommendations until a consensus is reached. When revisions are invited, a fact-based synthesis statement explaining their decision and outlining what is needed to prepare a revision will be listed below. The following reviewer(s) agreed to reveal their identity: Matthew Hearing.

Reviewer 1.

In this review, the authors address a timely issue in the field of behavioral neuroscience, namely the necessity of designing rigorous experiments that will allow for appropriate interpretation of scientific findings. The authors note that the advent of powerful technical tools, including optogenetics and calcium-imaging, has led to broad and sweeping conclusions about the attributions of cell populations or selective circuits to specific behavior, without considering that numerous other factors could also contribute to said behavior. I commend the authors on their initiative in writing this article and appreciate their willingness to start this discussion in the behavioral neuroscience field. I have several comments that should be addressed, however, before considering the manuscript appropriate for publication.

1. In the Abstract, the authors state “...subfields, similar assays are often used to support widely disparate conclusions which....direct disagreement with one another”. This statement was not directly addressed in the manuscript. Since this is one main impetus for the manuscript, it would behoove the authors to expand on this statement and perhaps provide specific examples. In particular, examples outside of the authors' laboratory and from existing literature would strengthen the authors' argument.

2. Similarly, in the Abstract, the authors state that they will discuss “common pitfalls... motivated behavior, with a focus on the most used approaches such as optogenetics, chemogenetics and calcium imaging.” With the exception of chemogenetics, the authors do not actually provide a discussion of such issues with these approaches. I think the authors should include common pitfalls and misconceptions that arise with the use of these techniques; perhaps the authors could touch on each of these approaches, commenting on best practices in experimental design and data interpretation.

3. The authors state that the use of unified and standardized behavioral controls could circumvent the issues that they discuss in the manuscript. This statement should be unpacked-are the authors suggesting that behavioral neuroscientists always use “task XX” and “task YY” as behavioral controls when studying “behavior ZZ” (i.e., standardized assays for the field)? Or are they suggesting that, as scientists, we just need to include control experiments to dissect behavioral outcomes/effects appropriately (i.e., not standardized, but appropriate for the particular experiment)? If the latter, this is not necessarily a new concept. Can the authors suggest ways in which we can improve on this?

4. Pg 8, line 173--”upmost” should be “utmost”

Reviewer 2

This review discusses basic principles and a need for more universal considerations that the behavioral neuroscience field should use when assessing data and how to best disseminate findings in a manner that can reduce spurious and/or often overgeneralized conclusions that often make inaccurate conclusions regarding causality. This was a very well written manuscript and while simplistic in its highlighting of interpretational pitfalls, it was a real pleasure to read. That said, the generalized approach to the discussion does somewhat reduce the impact. Below are a few considerations for the authors that, in my opinion, would boost the impact.

1. Overall, the manuscript reads as an opinion piece, with little inclusion of citations to provide specific examples of known misinterpretations, or at least examples where alternative interpretations could be made, outside of their own and other hypothetical ones. Perhaps this is to avoid singling out a given group of researchers? While nothing is inherently wrong with this, the authors do state “there is already a growing awareness of how often our claims of enural circuit function are incorrect or incomplete...”. Providing these examples would provide the reader an opportunity to see real-life situations that would likely help cement the considerations brought to light by the authors, especially if the misinterpretation was reasonably concluded.

2. There is discussion on the need to assess the effects of circuit manipulations on a multitude of behaviors to determine the extent and specificity of the effect. Additional discussion points on how to properly identify appropriate controls would be helpful as well.

3. The authors briefly touch on an increasingly transparent problem of publishing and funding structures driving the “need” for use of “glam” technologies and definitive conclusions. They also discuss throughout, a need to move towards more universal guidelines and increased discussion amongst the scientific community. I whole-heartedly agree with all of these, however it would be helpful if the authors could provide their thoughts on how to go about this in more detail.

## Author Response

Response to reviewers

eNeuro eN-OPN-0498-20

We would like to thank the reviewers for the thoughtful and insightful comments on the manuscript. They all noted that the manuscript was well written and that the issues addressed were timely. Reviewer 1 specifically noted “I commend the authors on their initiative in writing this article and appreciate their willingness to start this discussion in the behavioral neuroscience field.” Nevertheless, the reviewers identified multiple areas that could be improved as well as a number of helpful suggestions to make the manuscript clearer and more impactful. We have addressed these and other concerns in this revision, detailed below, and feel that with the incorporated revisions the manuscript has been much improved. We have noted new text and changes in the tracked manuscript.

Reviewer 1

Comment 1: In this review, the authors address a timely issue in the field of behavioral neuroscience, namely the necessity of designing rigorous experiments that will allow for appropriate interpretation of scientific findings. The authors note that the advent of powerful technical tools, including optogenetics and calcium-imaging, has led to broad and sweeping conclusions about the attributions of cell populations or selective circuits to specific behavior, without considering that numerous other factors could also contribute to said behavior. I commend the authors on their initiative in writing this article and appreciate their willingness to start this discussion in the behavioral neuroscience field. I have several comments that should be addressed, however, before considering the manuscript appropriate for publication.

Response: We want to thank the reviewer for the positive comments on this work.

Comment 2: In the Abstract, the authors state “...subfields, similar assays are often used to support widely disparate conclusions which....direct disagreement with one another”. This statement was not directly addressed in the manuscript. Since this is one main impetus for the manuscript, it would behoove the authors to expand on this statement and perhaps provide specific examples. In particular, examples outside of the authors' laboratory and from existing literature would strengthen the authors' argument.

Response: We apologize for the oversight and have now expanded upon this point within the manuscript. In the revised manuscript, we provide several specific examples, such as fear conditioning experiments where interpretations of neural control of freezing behavior are often attributed to memory when manipulations are performed in the hippocampus, while the same experiment when done on neural populations in the basolateral amygdala or other regions result in identical endpoints but are attributed to valence, threat, or fear. Some other examples are tasks looking a sucrose consumption where reduced sucrose consumption is attributed to anhedonia in the depression field, while similar reductions in sucrose consumption may be attributed to appetite in the feeding field. We made a deliberate decision to provide examples that are not tied to any individual manuscript, and therefore these examples are not provided with specific citations. This was done for two reasons. First, these examples are applicable to hundreds of citations. Second, we do not mean to criticize any individual laboratory's work. While the purpose of adding citations would be to enable the interested reader to go to the source material and identify first-hand how similar paradigms are differentially interpreted, we feel there is high likelihood that this might be misinterpreted by the cited laboratories.

Comment 3: Similarly, in the Abstract, the authors state that they will discuss “common pitfalls... motivated behavior, with a focus on the most used approaches such as optogenetics, chemogenetics and calcium imaging.” With the exception of chemogenetics, the authors do not actually provide a discussion of such issues with these approaches. I think the authors should include common pitfalls and misconceptions that arise with the use of these techniques; perhaps the authors could touch on each of these approaches, commenting on best practices in experimental design and data interpretation.

Response: We agree that the wording in the abstract suggested that the manuscript would include a more focused discussion of specific techniques. Our intention with this opinion piece was to start a discussion rather than provide an all-encompassing review of the literature with specific recommendations (that may differ across field, behavioral tasks, etc). We feel that adding too much additional discussion and expanding the piece would require this to then be a comprehensive review. In response to this comment as well as comments from Reviewer 2 we have removed this statement from the abstract, clarified our statement of purpose, and streamlined the manuscript to focus on our key points in a way that does not necessitate a comprehensive review of the circuit literature.

Comment 4: The authors state that the use of unified and standardized behavioral controls could circumvent the issues that they discuss in the manuscript. This statement should be unpacked-are the authors suggesting that behavioral neuroscientists always use “task XX” and “task YY” as behavioral controls when studying “behavior ZZ” (i.e., standardized assays for the field)? Or are they suggesting that, as scientists, we just need to include control experiments to dissect behavioral outcomes/effects appropriately (i.e., not standardized, but appropriate for the particular experiment)? If the latter, this is not necessarily a new concept. Can the authors suggest ways in which we can improve on this?

Response: We were not suggesting that people use the same experiments to approach questions. A diversity of approaches in a field is critical to understanding complex problems. That said, we do believe that reporting of findings from any given task should be fully standardized, and that the issue of methodology for reporting findings has been under emphasized in the literature. This is easily done by only talking about data in objective terms. For example, in discussing data from Pavlovian fear conditioning, one could say “manipulation X reduces freezing behavior” rather than “manipulation X disrupts memory”. In the first case it would be easy to unify data across fields as the conclusions are accurately and operationally described. In the second case the data are suggesting that a circuit controls memory, when reduced freezing could be driven by changes in valence, associative strength of the cue, motor response, or a number of other latent variables. We have reworded the manuscript to reflect this point more clearly.

Comment 5: Pg 8, line 173--“upmost” should be “utmost”

Response: We have made this change.

Reviewer #2

Comment 1: This review discusses basic principles and a need for more universal considerations that the behavioral neuroscience field should use when assessing data and how to best disseminate findings in a manner that can reduce spurious and/or often overgeneralized conclusions that often make inaccurate conclusions regarding causality. This was a very well written manuscript and while simplistic in its highlighting of interpretational pitfalls, it was a real pleasure to read. That said, the generalized approach to the discussion does somewhat reduce the impact. Below are a few considerations for the authors that, in my opinion, would boost the impact.

Response: We would like to thank the reviewer for the positive comments about the manuscript as well as the suggestions for improvement.

Comment 2: Overall, the manuscript reads as an opinion piece, with little inclusion of citations to provide specific examples of known misinterpretations, or at least examples where alternative interpretations could be made, outside of their own and other hypothetical ones. Perhaps this is to avoid singling out a given group of researchers? While nothing is inherently wrong with this, the authors do state “there is already a growing awareness of how often our claims of neural circuit function are incorrect or incomplete...”. Providing these examples would provide the reader an opportunity to see real-life situations that would likely help cement the considerations brought to light by the authors, especially if the misinterpretation was reasonably concluded.

Response: We apologize for the lack of clarity. This was intended to be an opinion piece and we have re-worded the abstract and streamlined the document to make this clear. In general, we have avoided calling out specific papers as being at fault given that this is an opinion piece and meant to be a reflection on the field at large (see response to Reviewer #1, Comment 2); however, if the reviewers or editors feel that specific citations should be provided, we would be willing to do so. We have also softened several statements throughout the manuscript to differentiate opinion vs objective claims.

Comment 3: There is discussion on the need to assess the effects of circuit manipulations on a multitude of behaviors to determine the extent and specificity of the effect. Additional discussion points on how to properly identify appropriate controls would be helpful as well.

Response: This is a great point, however, this discussion may be better suited for either a more comprehensive review or multiple manuscripts focused on specialized fields. What controls to run may change based on experimental design. We have now further clarified the purpose of the piece, and which aspects of experimentation we believe are practical to standardize across labs and fields (additionally, see response to Reviewer #1, Comment 4).

Comment 4: The authors briefly touch on an increasingly transparent problem of publishing and funding structures driving the “need” for use of “glam” technologies and definitive conclusions. They also discuss throughout, a need to move towards more universal guidelines and increased discussion amongst the scientific community. I whole-heartedly agree with all of these, however it would be helpful if the authors could provide their thoughts on how to go about this in more detail.

Response: With easy access to these technologies, now is the time to think about how we talk about our findings in a context that does not mislead or suggest our studies are more comprehensive than they are. The easiest thing we can do is to discuss our findings in operational terms. That is not to say that speculation is not important. Discussing what an experiment may mean in the context of the greater literature is an important aspect of moving science forward. However, being transparent with what was actually found and the definitive conclusions that can be made from that is an easy first step to allow for cross-field interpretation of findings and a greater understanding of how circuits are controlling behavior in different contexts and conditions. We have now clarified our thoughts within the manuscript and reflected them in our recommendations made at the end of the piece.
